# RETRACTION: LncRNA NEAT1/mir‐185‐5p/igf2 Axis Regulates the Invasion and Migration of Colon Cancer

**DOI:** 10.1002/mgg3.70247

**Published:** 2026-06-09

## Abstract

**RETRACTION**: S. Zhuang, Y. Cai, H. Liu, Y. Qin, and J. Wen, “LncRNA NEAT1/mir‐185‐5p/igf2 Axis Regulates the Invasion and Migration of Colon Cancer,” *Molecular Genetics & Genomic Medicine* 8, no. 4 (2020): e1125, https://doi.org/10.1002/mgg3.1125.

The above article, published online on 20 February 2020 in Wiley Online Library (wileyonlinelibrary.com), has been retracted by agreement between the authors; the journal Editor‐in‐Chief, Paraminder Dhillon; and Wiley Periodicals, LLC. The retraction has been agreed due to concerns raised by third parties. Specifically, image elements in Figures 1D, 3D, 3E, 4G, and 5C were identified as having been published elsewhere in a different scientific context. As a result, the editors consider the conclusions to be invalid. The authors were unable to adequately address these concerns, noting that, due to the time elapsed since the original publication, retrieval of the underlying raw data for verification would be difficult. The authors therefore requested the retraction of the article.

**RETRACTION**: S. Zhuang, Y. Cai, H. Liu, Y. Qin, and J. Wen, “LncRNA NEAT1/mir‐185‐5p/igf2 Axis Regulates the Invasion and Migration of Colon Cancer,” *Molecular Genetics & Genomic Medicine* 8, no. 4 (2020): e1125, https://doi.org/10.1002/mgg3.1125.

The above article, published online on 20 February 2020 in Wiley Online Library (wileyonlinelibrary.com), has been retracted by agreement between the authors; the journal Editor‐in‐Chief, Paraminder Dhillon; and Wiley Periodicals, LLC. The retraction has been agreed due to concerns raised by third parties. Specifically, image elements in Figures 1D, 3D, 3E, 4G, and 5C were identified as having been published elsewhere in a different scientific context. As a result, the editors consider the conclusions to be invalid. The authors were unable to adequately address these concerns, noting that, due to the time elapsed since the original publication, retrieval of the underlying raw data for verification would be difficult. The authors therefore requested the retraction of the article.

